# Systematically Study the Tensile and Compressive Behaviors of Diamond-like Carbon

**DOI:** 10.3390/nano13111772

**Published:** 2023-05-31

**Authors:** Jingxiang Xu, Yina Geng, Zhenhua Chu, Qingsong Hu, Yanhua Lei, Yang Wang

**Affiliations:** 1 College of Engineering Science and Technology, Shanghai Ocean University, Shanghai 201306, China; m200601287@st.shou.edu.cn (Y.G.); zhchu@shou.edu.cn (Z.C.); qshu@shou.edu.cn (Q.H.); 2 College of Ocean Science and Engineering, Shanghai Maritime University, Shanghai 201306, China; yhlei@shmtu.edu.cn; 3 Research Institute of Frontier Science, Southwest Jiaotong University, Chengdu 610031, China

**Keywords:** diamond-like carbon, mechanical properties, tensile, compression, molecular dynamic simulation

## Abstract

It is important to understand the mechanical properties of diamond-like carbon (DLC) for use not only in frictionand wear-resistant coatings, but also in vibration reduction and damping increase at the layer interfaces. However, the mechanical properties of DLC are influenced by the working temperature and its density, and the applications of DLC as coatings are limited. In this work, we systematically studied the deformation behaviors of DLC under different temperatures and densities using compression and tensile testing of DLC by molecular dynamics (MD) methods. In our simulation results, the values of tensile stress and compressive stress decreased and tensile strain and compressive strain increased as the temperature increased from 300 K to 900 K during both tensile and compressive processes, indicating that the tensile stress and tensile strain depend on the temperature. During the tensile simulation, Young’s modulus of DLC models with different densities had a different sensitivity to the increase in temperature, and the DLC model with a high density was more sensitive than that with a low density, which was not seen in the compression process. We conclude that the Csp3-Csp2 transition leads to tensile deformation, while the Csp2-Csp3 transition and relative slip dominate compressive deformation.

## 1. Introduction

Diamond-like carbon (DLC), as a new coating material with a different sp3 hybrid bond (sp2 hybrid bond) content, has unique and excellent mechanical properties such as high hardness, high chemical stability, friction resistance, and corrosion resistance, which can be widely used in anti-corrosion, mechanical, optoelectronic, biological engineering, and other applications [1,2,3,4]. DLC coatings are a key material in today’s hard disk drives (HDDs) because they protect disks and recording heads from corrosion and wear, acting as a barrier for disks [5]. DLC coatings can also be deposited on automotive components, which protects engine components from wear [6,7]. DLC coatings are also being considered for use in the aerospace sector to provide both mechanical protection and reliable low friction and wear (during ground testing and in environments other than Earth) [8]. A DLC coating, which can generate a constraint layer damping layer, has an optimal coating thickness, so the coating structure achieves the best balance between strength and damping capacity, and increasing the modulus difference between the coating and the substrate will improve the damping and stiffness of the coating system at the same time [9,10]. New multi-layer coating architectures can be investigated for thin-walled mechanical parts are being designed to reduce unwanted vibration behavior under high-speed operating conditions [11]. As a promising protective coating, the mechanical properties of DLC are of great importance [12]. However, the mechanical properties of DLC are influenced by the working temperature and its density, and the application of DLC to the coatings is limited.

In recent years, several studies investigated the mechanical properties of DLC [13,14,15,16,17,18,19,20]. These studies showed that annealing can influence mechanical properties [13,14,15,16]. For example, Peter et al. [15] used DC-PACVD to prepare DLC. It was found that the process of plastic deformation is accompanied by a decrease in stress, and with the increase in annealing temperature, the structural disorder decreases, and the graphite domain size increases. Wang et al. [16] explored the change in the mechanical properties of DLC film with temperature. They revealed that the internal stress of DLC will continuously decrease with increasing temperature, affecting the hardness and elastic modulus. Baimova et al. [17] analyzed the deformation behavior of diamond-like structures during hydrostatic tensile and compression, and the structural transformation was explained by the elongation of covalent bonds at high strain levels. The deformation mechanism of the mechanical properties of DLC with a density of 3.0 g/cm^3^ under uniaxial compression was investigated at high temperatures and low strain rates [18]. It was found that at high temperature diffusion relaxation is activated and dominates the structural changes in the DLC, inducing the Csp3-Csp2 transition, which leads to the decline of its mechanical properties. Bai et al. [19] analyzed the deformation mechanism of the mechanical behavior of DLC with a density of 3.02 g/cm^3^ at 300 K under a uniaxial tensile test and found that with increasing tensile strain, the fraction of Csp3 decreased and the fraction of Csp2 increased. Our previous study revealed the relationship between Young’s modulus of DLC and the density and proposed quantitative relations among the effective coordination number, density, and Young’s modulus [20]. These studies have adequately investigated the mechanical property deformation mechanism of DLC, but they only analyze DLC with a certain density or temperature. The mechanism for the effect of temperature and density on the mechanical properties of DLC remains unknown. In particular, as a coating, DLC is subjected to tensile and compressive stresses. Thus, it is essential to study the different deformation mechanisms of DLC with temperature and density under tensile and compressive stresses.

In this work, uniaxial compression and tensile testing of DLC were simulated using molecular dynamics (MD) methods to study the deformation behaviors under different temperatures and densities. The changes in the mechanical properties of DLC were investigated. The mechanism for the effect of the different temperatures and densities on the deformation behaviors is discussed here. Finally, the difference in the deformation behaviors under the tensile and compressive stresses is revealed.

## 2. Computational Details

Molecular dynamics (MD) simulation was used to investigate the deformation behaviors in this study because the MD method can analyze and define the properties of materials at the microstructural level that cannot be characterized by macroscopic experiments [21,22,23]. A reactive force field (ReaxFF), a new generation of molecular dynamics methods developed based on quantum mechanics and bond-level functions, was employed in order to describe the DLC structures. ReaxFF has been shown to be a good force field for simulating doping, stretching, and compression [24,25,26]. The description of ReaxFF can be found in previous studies [27,28]. In our previous study, we developed a transferable ReaxFF parameter set for carbon- and silicon-based solid systems, and this parameter set can describe the DLC well [29]. Thus, we used the same parameter set, and all the MD simulations used in this study were executed by our developed code LASKYO [30].

**Figure 1:**
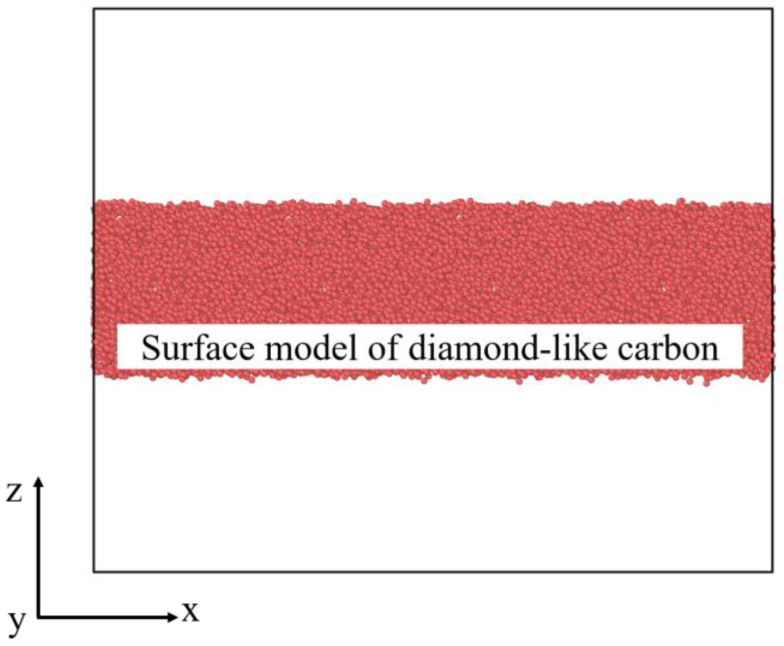
Simulation model of DLC (120.45 Å × 30.21 Å × 100.0 Å).

In this study, the models of the DLC with different densities were built by using the quick quenching method [31,32], which is a common method to construct a DLC model. In this generation process, a diamond crystal structure is created, and periodic boundary conditions are applied in the three directions of x, y, and z. The diamond structure is first heated by using the NVT (constant number of atoms, volume, and temperature) ensemble from 300 K to 8000 K above the melting point of the diamond. Then, the models are quenched in 10 ps to obtain the amorphous phase, which reduces the temperature from 8000 K to 300 K. Next, the annealed models are relaxed with the NVT ensemble for 25 ps and then with the NPT ensemble (constant number of atoms, pressure, and temperature) for 25 ps to eliminate the residual stress. By using the above method, DLC models with different densities were constructed by changing the initial density of the simulation model. In order to study the effect of the density on the mechanical properties of DLC, DLC models with densities of 2.34 g/cm^3^, 2.60 g/cm^3^, 2.87 g/cm^3^, and 3.01 g/cm^3^ were built. All DLC bulk models contained 4096 atoms. Next, we built the surface models of DLC based on the DLC bulk models and expanded them by a factor of 4 in the x-direction in order to investigate the fracture behaviors. The number of C atoms increased from 4096 to 16,394 and the periodic boundary conditions were only applied in the directions of x and y for the surface models. Then, the surface models were relaxed for 10 ps at 300 K temperature. The surface model of DLC with a density of 3.01 g/cm^3^ in this study is shown in Figure 1. The actual dimensions of the simulation box are 120.45 Å, 30.21 Å, and 100.0 Å. The actual dimensions of the surface are 120.45 Å, 30.21 Å, and 30.02 Å.

For the structural analysis, we used bond order to check the bond between two atoms. Then, the DLC surface model was stretched and compressed along the x-direction while the cell was relaxed along the y-direction and fixed along the z-direction. Young’s modulus was obtained as the slope of the stress–strain curve in the linear elastic range.

In the uniaxial tensile and compression simulations, the strain rate was set as 0.01 ps^−1^, which is the same as in the previous studies [19,33]. The maximum strains applied along the x-axis in uniaxial tension and compression were 2 and 0.5, respectively. Finally, the mechanical properties of DLC models with different densities were compared and analyzed. To analyze the effect of temperature on the mechanical properties, the working temperatures were 300 K, 500 K, 700 K, and 900 K. During the tensile and compression processes, the controlling temperature of the systems was obtained by the NPT ensemble with the 0.25 fs step. Considering the influence of temperature on mechanical properties, the Nose–Hoover temperature control method was adopted in the NPT ensemble to keep the system at 300 K, 500 K, 700 K, and 900 K, with relaxation at 170 ps and 50 ps to simulate the real operating temperature during the tensile and compression processes.

## 3. Results and Discussion

### 3.1. Tensile Properties of DLC

**Figure 2:**
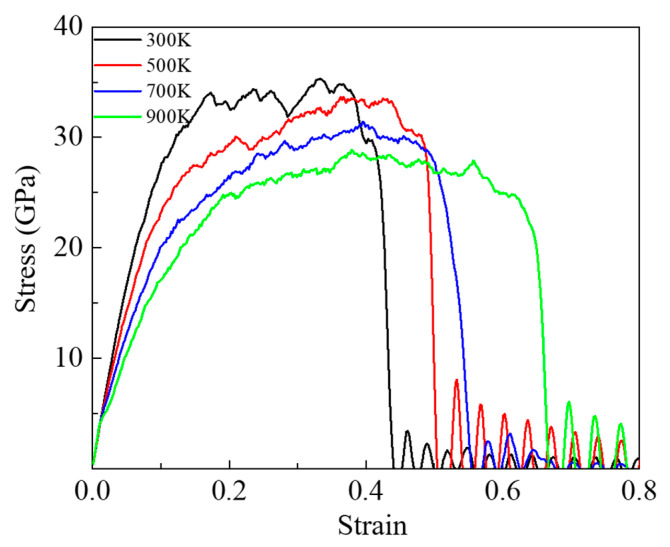
Stress–strain curves of DLC surface models with the density of 2.87 g/cm^3^ at different temperatures under tensile process.

We first performed MD tensile simulations of the DLC models with different densities to investigate the effect of temperature and density on the mechanical properties during the tensile process. Figure 2 presents the stress–strain curve of the DLC model with a density of 2.87 g/cm^3^ at different working temperatures. When the temperature increases, we can see that the stress–strain curve changes significantly. In the case of 300 K, the curve reached a maximum stress value of 35.30 GPa at a strain of 0.33. Here, we defined the maximum stress as the tensile stress and the corresponding strain as the tensile strain [34]. As the temperature increases from 300 K to 900 K, the values of tensile stress decrease, and the corresponding strain increases. This is indicating that the tensile stress and corresponding strain depend on the temperature, and the brittle–plastic transformation is obvious. At higher temperatures, the plastic deformation of DLC is more obvious. The tensile simulations of the DLC models with densities of 2.34, 2.60, and 3.01 g/cm^3^ were also executed, and the results are shown in Figure S1. We observed a similar trend in the tensile stress with increasing temperature at each density.

**Figure 3:**
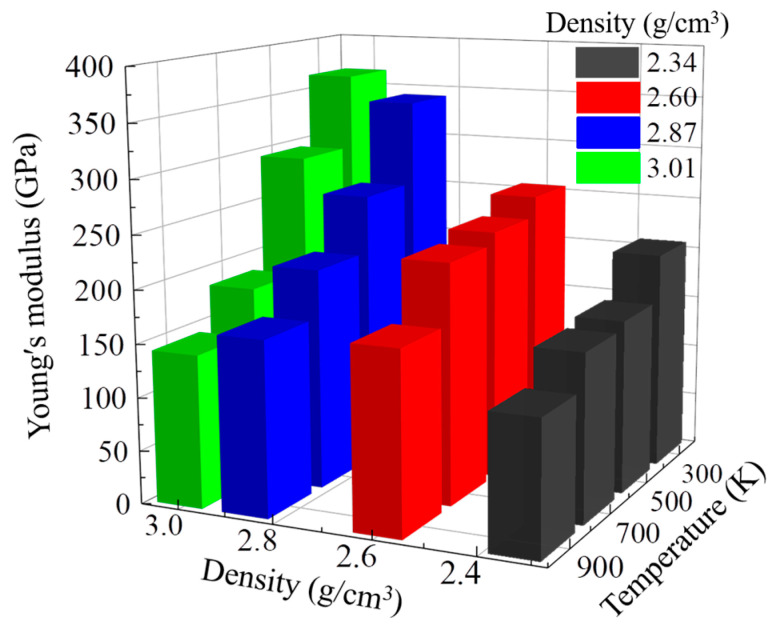
Changes in Young’s modulus as a function of the density and temperature in the model with densities of 2.34, 2.60, 2.87, and 3.01 g/cm^3^ under tensile simulation.

Figure 3 shows the dependence of Young’s modulus on temperature and density. Young’s modulus is one of the important macroscopic mechanical properties of DLC and is a parameter to measure the resistance of materials to deformation. At 300 K, Young’s modulus of the DLC model increases with the increase in density, which was also reported by Schultrich [35]. When the temperature is higher than 500 K, Young’s modulus first increases and then decreases with increasing density. Meanwhile, for the same density, we found that Young’s modulus decreases with increasing temperature. Previous studies also reported that with increasing temperature, Young’s modulus of DLC decreases due to the increase in hydrogen content in the film leading to a decrease in the density of the film [36,37]. Interestingly, with increasing temperature, Young’s modulus of DLC models decreases from 206.75 to 124.99 GPa at low densities; however, Young’s modulus decreases from 365.48 to 143.24 GPa at high densities. This indicates that Young’s modulus of DLC models with different densities has a different sensitivity to the increase in temperature. The DLC model with a high density is more sensitive than that with a low density.

**Figure 4:**
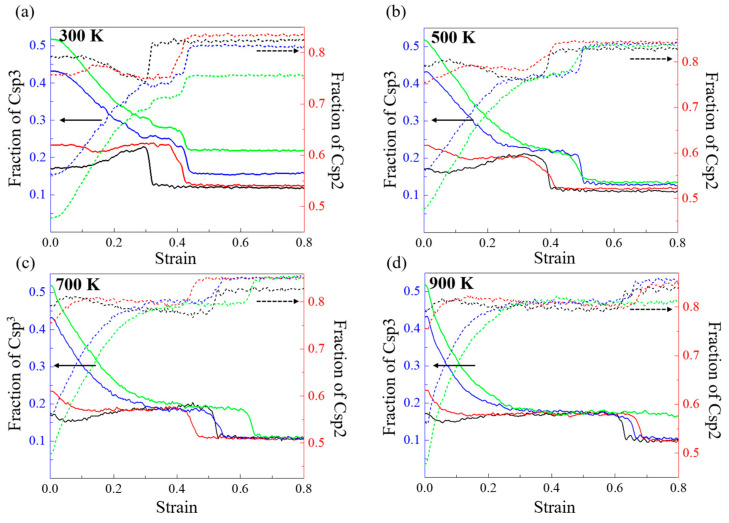
Change in fractions of Csp3 and Csp2 in the DLC model with densities of 2.34, 2.60, 2.87, and 3.01 g/cm^3^ at (**a**) 300 K, (**b**) 500 K, (**c**) 700 K, and (**d**) 900 K under tensile process. Black, red, blue, and green lines indicate the DLC model with densities of 2.34, 2.60, 2.87, and 3.01 g/cm^3^, respectively. Solid and dashed lines are fractions of Csp3 and Csp2, respectively. (The solid arrow represents the solid line in the figure, which is based on the left vertical axis scale, and the dashed arrow represents the dashed line in the figure based on the right vertical axis scale.).

Then, to investigate the mechanism of the change in Young’s modulus with the different densities and temperatures in the model with densities of 2.34, 2.60, 2.87, and 3.01 g/cm^3^, we quantified the contents of Csp3 and Csp2 atoms for monitoring the evolution of DLC structure because the mechanical properties of DLC are mainly changed by adjusting the content of Csp3 hybrid bonds in the film structure [36]. Figure 4 shows the change in the fractions of Csp3 and Csp2 in the DLC model with densities of 2.34, 2.60, 2.87, and 3.01 g/cm^3^ at different working temperatures. As shown in Figure 4a, at 300 K, the fractions of the Csp3 atoms in the DLC model with densities of 2.34, 2.60, 2.87, and 3.01 g/cm^3^ at the strain of 0 are 0.17, 0.23, 0.43, and 0.52, respectively, and the fractions of the Csp2 atoms in the DLC model with densities of 2.34, 2.60, 2.87, and 3.01 g/cm^3^ at the strain of 0 are 0.79, 0.76, 0.56, and 0.48, respectively. This indicates that the higher the density of DLC, the higher the fraction of Csp3. This is why the DLC model with a high density has a higher Young’s modulus. Then, the fraction of Csp3 decreases with increasing tensile strain during the tensile process; however, the fraction of Csp2 also keeps increasing with increasing tensile strain, indicating that the fraction of Csp3 atoms in the DLC gradually transforms to Csp2 with stretching. Then, we observe that the fraction of Csp3 drops dramatically and gradually tends toward equilibrium at strains of 0.33, 0.43, 0.45, and 0.44, with continued stretching. The strain value to reach equilibrium corresponds to the strain value to failure strain. Meanwhile, the fractions of Csp3 in the DLC model with densities of 2.34, 2.60, 2.87, and 3.01 g/cm^3^ are 0.12, 0.13, 0.16, and 0.22, respectively, at the point of film failure. This indicates that the decrease in the fraction of Csp3 at a high density is larger than that at a low density. There is a similar trend in the fractions of Csp3 and Csp2 with increasing density of the DLC model at 500, 700, and 900 K. In addition, as shown in Figure 4b–d, the rate of descent of Csp3 becomes greater during the tensile process with increasing temperature. This explains why Young’s modulus at high densities in Figure 3 decreases faster and is more sensitive to temperature.

### 3.2. Compressive Properties of DLC and Schemes

**Figure 5:**
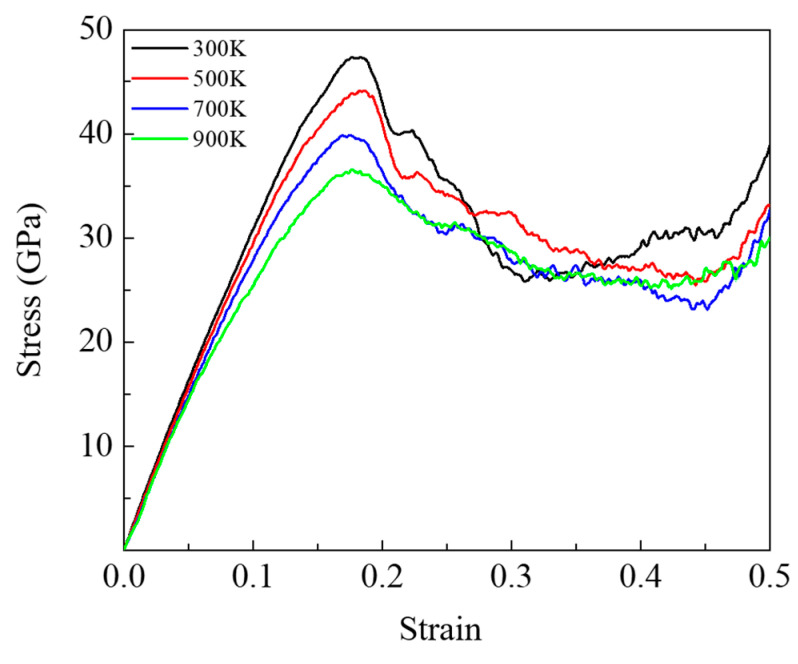
Stress–strain curves of DLC surface models with the density of 2.87 g/cm^3^ at different temperatures under compressive process.

Then, we investigated the effect of temperature and density on the mechanical properties during the compressive process. Figure 5 shows the stress–strain curve of the DLC model with a density of 2.87 g/cm^3^ at different temperatures. Similarly, in the case of 300 K, the curve reached a maximum stress value of 47.34 GPa at a strain of 0.18. Here, we defined the maximum stress as the compressive stress and the corresponding strain as the compressive strain. At 300 K, the stress increases with the increasing compressive strain, and then the stress increases after reaching a stress of 47.34 GPa. This indicates that the DLC model with a density of 2.87 g/cm^3^ produced high stress. As the temperature increases from 300 K to 900 K, the values of compressive stress decrease from 47.34 to 36.59 GPa, and the compressive strain shows little change. This is indicating that the compressive stress depends on the temperature and that temperature has a small effect on the compressive strain. Compressive simulations of the DLC models with densities of 2.34, 2.60, and 3.01 g/cm^3^ were also performed, and we observed a similar trend in the compressive stress with increasing temperature at each density, as shown in Figure S2.

**Figure 6:**
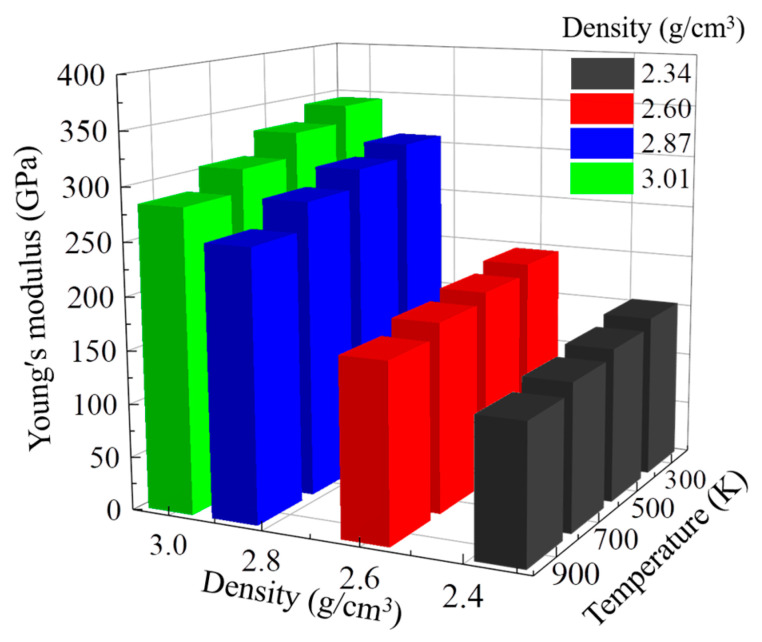
Changes in Young’s modulus as a function of the density and temperature in the model with densities of 2.34, 2.60, 2.87, and 3.01 g/cm^3^ under compressive simulation.

Figure 6 shows the dependence of Young’s modulus under compression on temperature and density. At the same density, Young’s modulus decreases slightly with increasing temperature. During the compression process, we found that Young’s modulus of different densities decreased more evenly at high temperatures, in that when the temperature is 900 K, Young’s modulus of 2.34, 2.60, 2.87, and 3.01 g/cm^3^ decrease by 16.12%, 15.29%, 17.67%, and 17.44%, respectively, which was different from the larger variation during the tensile process. This indicates that temperature has a smaller effect on Young’s modulus during compression, unlike the tensile results. Moreover, Young’s modulus increases with increasing density due to the increased Csp3 content. It is interesting to note that Young’s modulus was not seen to have different sensitivities to the increase in temperature for models with different densities during compression.

**Figure 7:**
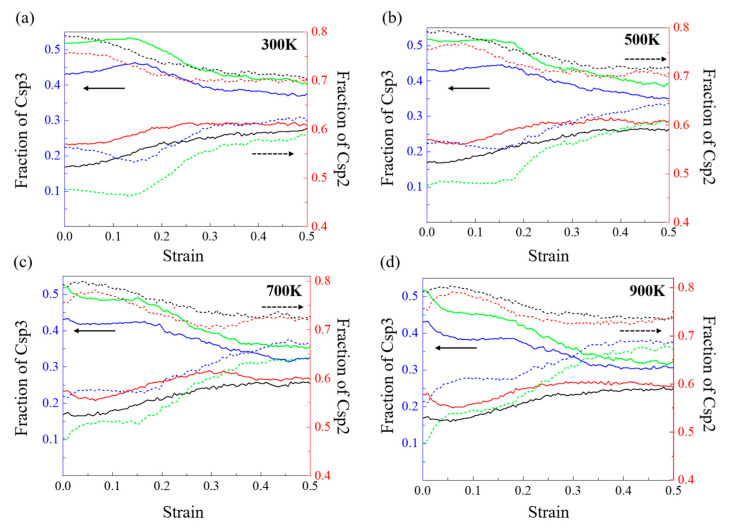
Change in fractions of Csp3 and Csp2 in the DLC model with densities of 2.34, 2.60, 2.87, and 3.01 g/cm^3^ at (**a**) 300 K, (**b**) 500 K, (**c**) 700 K, and (**d**) 900 K under compressive process. Black, red, blue, and green lines indicate the DLC model with densities of 2.34, 2.60, 2.87, and 3.01 g/cm^3^, respectively. Solid and dashed lines are fractions of Csp3 and Csp2, respectively (The solid arrow represents the solid line in the figure, which is based on the left vertical axis scale, and the dashed arrow represents the dashed line in the figure based on the right vertical axis scale.).

Figure 7 shows how the fraction of Csp3 and Csp2 atoms in the DLC models with densities of 2.34, 2.60, 2.87, and 3.01 g/cm^3^ varies at different operating temperatures. As shown in Figure 7a, at 300 K, the fractions of the Csp3 and Csp2 atoms in the DLC model with densities of 2.34, 2.60, 2.87, and 3.01 g/cm^3^ at the strain of 0 are the same as in Figure 4a. As shown in Figure 7a, at 300 K, the fraction of Csp3 atoms in all models starts to increase with compression. This is because the density increases with compression due to the decrease in the volume. In addition, we see less variation in the fraction of Csp3 atoms in the DLC models during compression, in contrast to the more variable stretching results. Meanwhile, the fraction of Csp3 atoms in the DLC models with densities of 2.87 and 3.01 g/cm^3^ increases slightly before reaching a maximum, while the DLC models with densities of 2.34 and 2.60 g/cm^3^ show a continuous increase in the fraction of Csp3 atoms. In addition, the fraction of Csp2 atoms decreases with increasing compressive strain at the initial stage, suggesting that the fraction of Csp2 atoms in the DLC gradually transforms to Csp3 with compression. With continued compression, the fraction of Csp2 atoms in the DLC models with densities of 2.87 and 3.01 g/cm^3^ increases at a slower rate towards equilibrium. The fraction of Csp2 atoms in the DLC models with densities of 2.34 and 2.60 g/cm^3^ starts to increase after the failure of the DLC model, indicating the microstructural rearrangement. Furthermore, as shown in Figure 7b–d, the rate of decrease in the fraction of Csp2 atoms slows down with increasing temperature during compression, and at 900 K, it appears that the fraction of Csp2 initially increases with compression. This is due to the combination of the Csp2-Csp3 transition during compression and the increase in the fraction of Csp2 due to the increase in temperature.

**Figure 8:**
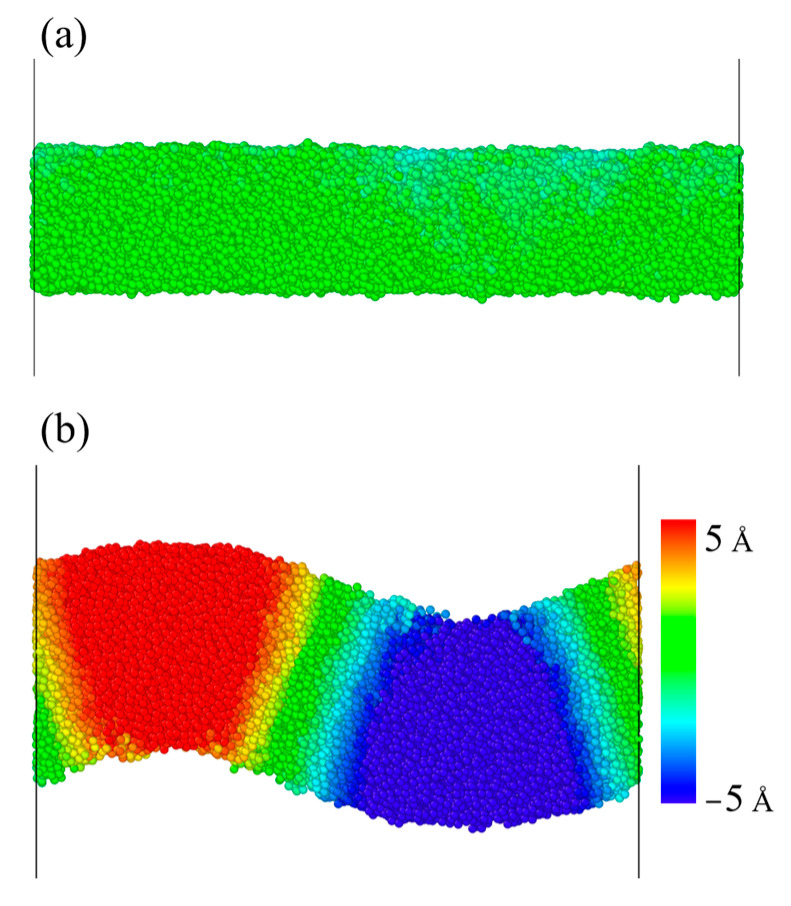
The snapshots of atomic displacements along the z-axis of the DLC model with the density of 3.01 for (**a**) tensile and (**b**) compressive simulation at strains of 0.2.

To investigate the deformation mechanism under the tensile and compressive processes, we calculated the displacement of every atom along the z-axis, representing the degree of relative slip. The contour map after the yielding of the DLC model with the density of 3.01 g/cm^3^ under tensile and compressive processes is shown in Figure 8. In our simulation results, the displacement of each atom is close to zero for the stretching process, whereas some of the atoms are displaced for the compressive process, indicating that a slip has occurred. During compression, relative slip occurs after film failure. These results show that the deformation mechanism is different during the tensile and compressive processes. After the compression failure of the films, their sp3-sp2 transition is closely related to the relative slip. During the compression process, the relative slip after the failure of the film accelerates the structural transformation, while when the same strain and ambient temperature are in the stretching process, no relative slip occurs in the z-direction, indicating that the failure deformation behavior of DLC in the compressive and tensile process is different.

## 4. Conclusions

In this study, to understand the mechanical properties of diamond-like carbon (DLC), we systematically studied the deformation behaviors of DLC under different temperatures and densities using tensile and compression testing of DLC by molecular dynamics (MD) methods. In our simulation results, the values of tensile stress decreased and tensile strain increased as the temperature increased from 300 K to 900 K during tensile processes, indicating that the tensile stress and tensile strain depend on the temperature. At higher temperatures, the plastic deformation of DLC is more pronounced. During compressive processes, the values of compressive stress decrease, and the compressive stress and strain also slightly increase as the temperature increases from 300 K to 900 K, indicating that the compressive strain depend on the temperature, and that temperature has a small effect on compressive stress and strain.

Higher-density DLCs exhibit better mechanical properties and are more sensitive to temperature. During the tensile simulation, Young’s modulus of DLC models with different densities had a different sensitivity to the increase in temperature, and the DLC model with a high density was more sensitive than that with a low density, which was not seen in the compression process. We conclude that the Csp3-Csp2 transition leads to tensile deformation, while the Csp2-Csp3 transition and relative slip lead to compressive deformation.

## Data Availability

All data needed to evaluate the conclusions in the paper are present in the paper and the Supplementary Materials.

## Supplementary Materials

The following supporting information can be downloaded at: https://www.mdpi.com/article/10.3390/nano13111772/s1, Figure S1: Stress–strain curves of DLC surface models with densities of (a) 2.34 g/cm^3^, (b) 2.60 g/cm^3^, and (c) 3.01 g/cm^3^ at different temperatures under tensile process; Figure S2: Stress–strain curves of DLC surface models with densities of (a) 2.34 g/cm^3^, (b) 2.60 g/cm^3^, and (c) 3.01 g/cm^3^ at different temperatures under compressive process.
