# Supplementary material for: Systematically Study the Tensile and Compressive Behaviors of Diamond-like Carbon
Source: Nanomaterials (Basel). 2023 May 31;13(11):1772. doi: 10.3390/nano13111772 (PMC10254679; doi:10.3390/nano13111772)
Supplement: Supplementary file 1 [file nanomaterials-13-01772-s001.zip › nanomaterials-2419018-supplementary.pdf]

Supporting Information for

Systematically Study the Tensile and Compressive Behaviors of Diamond-like Carbon

Jingxiang Xu [1,*], Yina Geng [1], Zhenhua Chu [1], Qingsong Hu [1], Yanhua Lei [2] and Yang Wang [3,*]

[1] College of Engineering Science and Technology, Shanghai Ocean University, Shanghai 201306, China; m200601287@st.shou.edu.cn (Y.G.); zhchu@shou.edu.cn (Z.C.); qshu@shou.edu.cn (Q.H.)

[2] College of Ocean Science and Engineering, Shanghai Maritime University, Shanghai 201306, China; yhlei@shmtu.edu.cn

[3] Research Institute of Frontier Science, Southwest Jiaotong University, Chengdu 610031, China

* Correspondence: jxxu@shou.edu.cn (J.X.); yang.wang@swjtu.edu.cn (Y.W.)

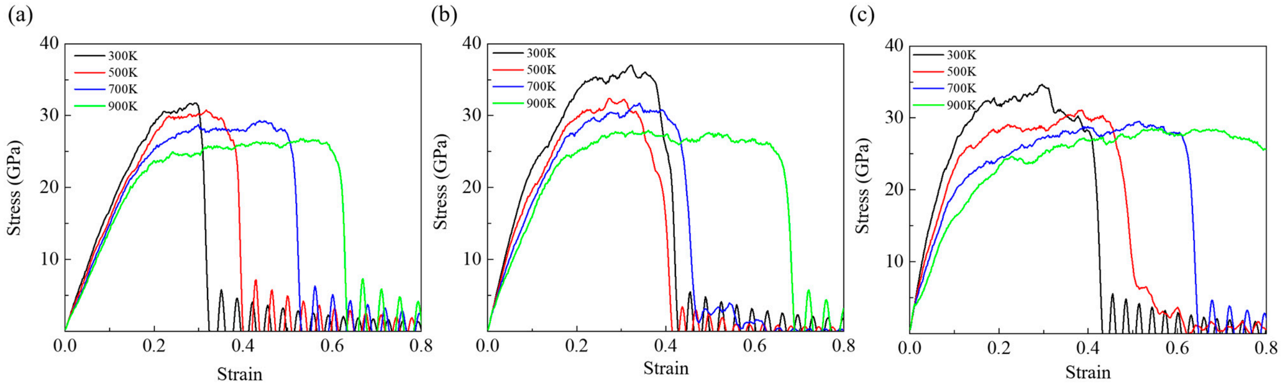

Figure S1. Stress-strain curves of DLC surface models with the density of (a) 2.34 g/cm^3, (b) 2.60 g/cm^3, and (c) 3.01 g/cm^3 at different temperature under tensile process.

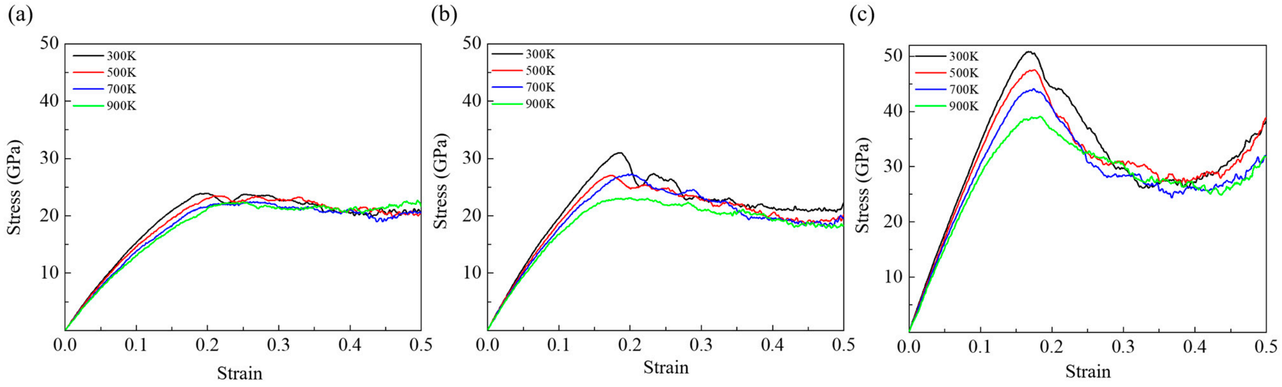

Figure S2. Stress-strain curves of DLC surface models with the density of (a) 2.34 g/cm^3, (b) 2.60 g/cm^3, and (c) 3.01 g/cm^3 at different temperature under compressive process.
